# Rivastigmine for ECT-induced cognitive adverse effects in late life depression (RECALL study): A multicenter, randomized, double blind, placebo-controlled, cross-over trial in patients with depression aged 55 years or older: Rationale, objectives and methods

**DOI:** 10.3389/fpsyt.2022.953686

**Published:** 2022-07-15

**Authors:** Marieke J. Henstra, Thomas C. Feenstra, Rob M. Kok, Harm-Pieter Spaans, Eric van Exel, Annemiek Dols, Mardien Oudega, Anton C. M. Vergouwen, Adriano van der Loo, Pierre M. Bet, Stephan A. Loer, Merijn Eikelenboom, Pascal Sienaert, Simon Lambrichts, Filip Bouckaert, Judith E. Bosmans, Nathalie van der Velde, Aartjan T. F. Beekman, Max L. Stek, Didi Rhebergen

**Affiliations:** ^1^Department of Internal Medicine and Geriatrics, Amsterdam UMC location AMC, University of Amsterdam, Amsterdam, Netherlands; ^2^Department of Clinical Epidemiology, Biostatistics and Bioinformatics, Amsterdam UMC location AMC, University of Amsterdam, Amsterdam, Netherlands; ^3^Aging and Later Life Program, Amsterdam Public Health Research Institute, Amsterdam, Netherlands; ^4^Amsterdam Public Health Research Institute, Mental Health Program, Amsterdam, Netherlands; ^5^Department of Research, GGZ Centraal Mental Health Care, Amersfoort, Netherlands; ^6^Department of Old Age Psychiatry and ECT Center Haaglanden, Parnassia Psychiatric Institute, The Hague, Netherlands; ^7^Department of Research, GGZ inGeest Mental Health Care, Amsterdam, Netherlands; ^8^Department of Psychiatry, Amsterdam UMC location Vrije Universiteit Amsterdam, Amsterdam, Netherlands; ^9^Amsterdam Neuroscience, Mood, Anxiety, Psychosis, Sleep and Stress Program, Amsterdam, Netherlands; ^10^Amsterdam Neuroscience, Neurodegeneration Program, Amsterdam, Netherlands; ^11^Department of Psychiatry and Medical Psychology, OLVG Hospital, Amsterdam, Netherlands; ^12^Department of Clinical Pharmacology and Pharmacy, Amsterdam UMC location VUmc, Amsterdam, Netherlands; ^13^Department of Anesthesiology, Amsterdam UMC location VUmc, Amsterdam, Netherlands; ^14^Department of Neurosciences, University Psychiatric Center KU Leuven, Academic Center for ECT and Neuromodulation (AcCENT), KU Leuven, Leuven, Belgium; ^15^Department of Geriatric Psychiatry, University Psychiatric Center KU Leuven, KU Leuven, Leuven, Belgium; ^16^Department of Health Sciences, Faculty of Science, Vrije Universiteit, Amsterdam, Netherlands

**Keywords:** electroconvulsive therapy (ECT), late life depression (LLD), cognitive side effects, interictal delirium, rivastigmine, cholinesterase inhibitor

## Abstract

**Background:**

Cognitive side-effects are an important reason for the limited use of electroconvulsive therapy (ECT). Cognitive side-effects are heterogeneous and occur frequently in older persons. To date, insight into these side-effects is hampered due to inconsistencies in study designs and small sample sizes. Among all cognitive side-effects, confusion and delirious states are especially troublesome for patients, relatives and clinicians. In particular inter-ictal delirium-like states are worrisome, since they may lead to premature treatment discontinuation. Besides a need for further insight into determinants of cognitive side-effects of ECT, there is a great need for treatment options.

**Methods and design:**

The Rivastigmine for ECT-induced Cognitive Adverse effects in Late Life depression (RECALL) study combines a multicenter, prospective cohort study on older patients with depression, treated with ECT, with an embedded randomized, placebo-controlled cross-over trial to examine the effect of rivastigmine on inter-ictal delirium. Patients are recruited in four centers across the Netherlands and Belgium. We aim to include 150 patients into the cohort study, in order to be able to subsequently include 30 patients into the trial. Patients are included in the trial when inter-ictal delirium, assessed by the Confusion Assessment method (CAM), or a drop in Mini Mental State Examination (MMSE) score of ≥4 during ECT, develops. In the cohort study, comprehensive measurements of ECT-related cognitive side-effects—and their putative determinants—are done at baseline and during the ECT-course. The primary outcome of the clinical trial is the effectiveness of rivastigmine on inter-ictal delirium-severity, assessed with a change in the Delirium Rating Scale-Revised-98. Secondary outcomes of the clinical trial are several ECT-characteristics and side-effects of rivastigmine.

**Discussion:**

This study is the first clinical trial with a focus on ECT-induced, inter-ictal delirium. The cohort provides the basis for recruitment of patients for the cross-over trial and additionally provides an excellent opportunity to unravel cognitive side-effects of ECT and identify putative determinants. This paper describes the rationale and study protocol.

**Clinical trial registration:**

EudraCT 2014-003385-24.

## Introduction

Electroconvulsive therapy (ECT) is considered safe and the most effective treatment for major depressive disorder (1). Despite these qualifications, the use of ECT in clinical practice around the world is limited. This can be attributed to, amongst others, fear of cognitive side-effects, which include amnesia, confusional- or delirious states (e.g., postictal disorientation/-agitation, and prolonged postictal delirium or inter-ictal delirium), and other non-memory cognitive side-effects (e.g., executive function disorder) (2–6). Cognitive side-effects are common in ECT-patients, with 41% reporting anterograde memory impairment, confusion in 37%, and post-ECT delirium in 5.7–12% of patients (7–11). To date, insight into these side-effects is hampered due to a lack of consensus on cognitive testing during and after ECT and variable ECT techniques across studies. This resulted in large heterogeneity in (timing of) outcome measures between studies (12, 13).

Of all studies on cognitive side-effects in ECT, studies on delirious states following ECT have been relatively sparse, in comparison to, for example, memory related side-effects (5, 10). This is troublesome, as especially the inter-ictal (or prolonged post-ictal) delirious states (henceforth called inter-ictal delirium) greatly burden patients and their relatives, and are an important reason to (prematurely) stop ECT, resulting in failure to achieve remission. As ECT is often the last treatment option for severe depression, failure to achieve remission with ECT may lead to persistent loss of quality of life, institutionalization, and high societal costs. It is currently not known how to treat inter-ictal delirium, as non-medication interventions, benzodiazepines, and antipsychotic medication are seldom effective. However, some reports have shown promising results for (acetyl)cholinesterase inhibitors (AChI) to treat inter-ictal delirium specifically (6, 14).

The rational to supplement acetylcholine in the treatment of post-ictal delirium is based on the hypothesis that changes in acetylcholine levels could be associated with cognitive side-effects of ECT (15). This may particularly involve older persons, considering changes in cholinergic signaling in the aging brain (16). Additionally, a lack of acetylcholine—the most important neurotransmitter of the cholinergic system—is thought to be one of the important causes of delirium in general (17). It is known that ECT initially causes an ictal peak of acetylcholine and postictal (e.g., post-ECT) drop of acetylcholine, mediated by cholinesterase (15, 18, 19). In 2015, van Schaik et al. published a case-series describing the putative beneficial effect of transdermal rivastigmine on ECT-induced cognitive side-effects in older ECT patients (8). Hence, a systematic review was done to examine the effect of (acetyl)cholinesterase inhibitors (AChI) (e.g., rivastigmine, donepezil, galantamine) to reduce cognitive side-effects of ECT (20). This systematic review by Henstra et al., included 5 clinical trials which all showed a positive effect of AChI on cognitive tests, but none of these studies studied the post-ECT delirium or inter-ictal delirium.

The primary objective of this study is to investigate in a multicenter, randomized, double-blind, placebo-controlled cross-over trial, whether rivastigmine can be used as a novel treatment to treat ECT-induced inter-ictal delirium in older persons. Additionally, we aim to investigate side-effects of rivastigmine, including its effects on ECT parameters and anesthesia-induced muscle relaxation. In order to detect persons with inter-ictal delirium who are eligible for the trial, longitudinal follow-up of persons treated with ECT is needed. Therefore, we embedded the trial in a cohort study. In this cohort study, comprehensive measurements of ECT-related cognitive side-effects—and their putative determinants—are done to gain insight into the broad palette of cognitive side-effects of ECT.

## Methods and analysis

In line with the SPIRIT (Standard Protocol Items: Recommendations for Interventional Trials) guidelines, we provide information on scientific background, the rationale and objectives, the design and methodology of this study (21).

### Study design and research setting

**Figure 1:**
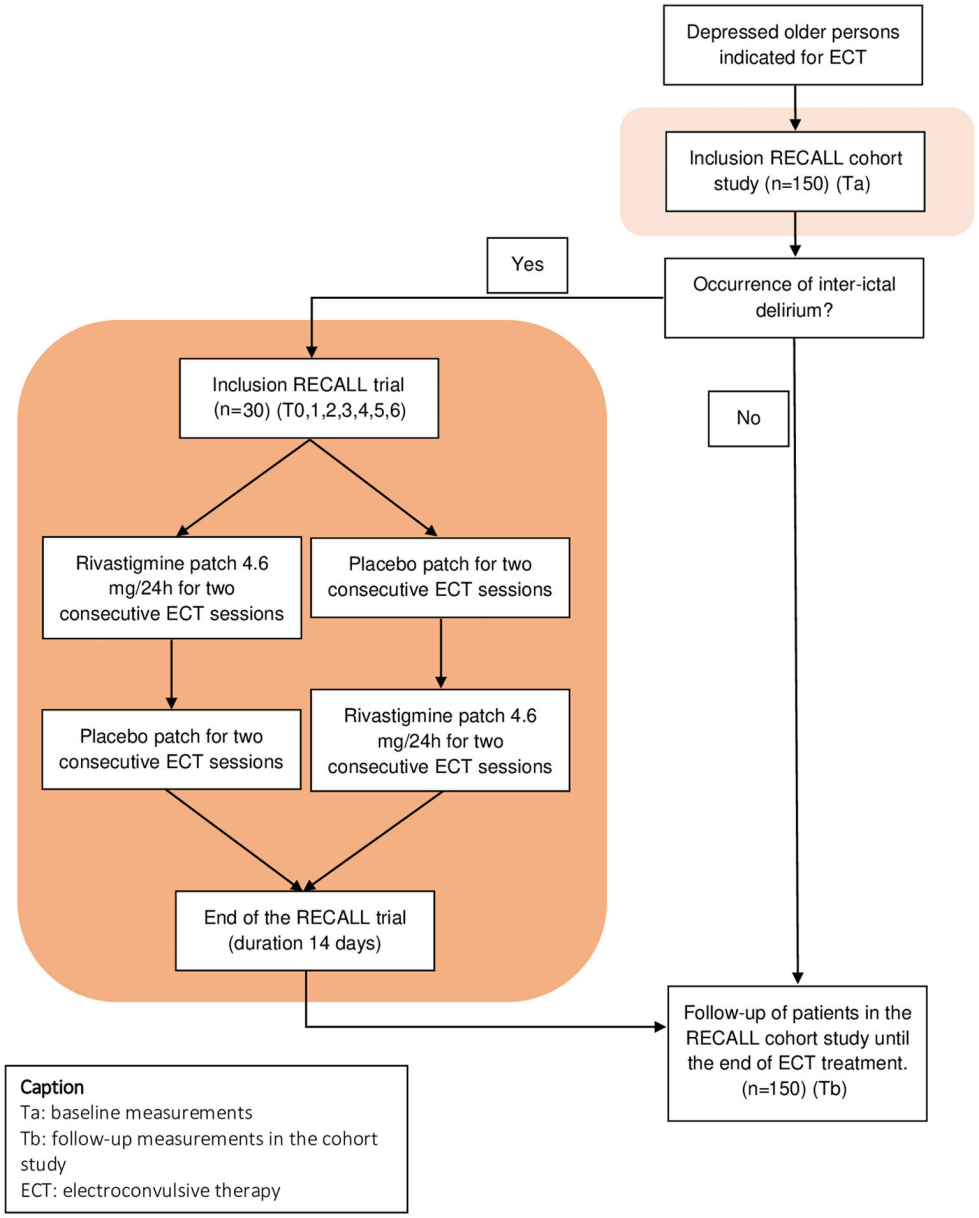
Schematic representation of RECALL study.

The RECALL study combines a multicenter, prospective cohort study on older patients with depression who are referred for ECT to study the broad palette of cognitive side-effects (and their determinants), with an embedded cross-over trial to examine rivastigmine as a treatment option for patients with inter-ictal delirium (see Figure 1 for a schematic overview of the RECALL study). The trial is designed as a randomized, double blind, placebo-controlled, cross-over trial. Patients are recruited at four mental healthcare institutes; (1) GGZ inGeest (partner-institution of Amsterdam University Medical Center, formerly VU University Medical Center), Amsterdam, The Netherlands, (2) Parnassia Psychiatric Institute, The Hague, The Netherlands, (3) OLVG-West, Amsterdam, The Netherlands, and (4) UPC KU Leuven, Leuven, Belgium. GGZ InGeest, and Parnassia Psychiatric Institute are general mental healthcare institutes; OLVG West is a general hospital with a psychiatric ward, and UPC KU Leuven is an academic hospital.

### Study population and inclusion criteria

In- and outpatients, aged 55 years or older, fulfilling the Composite International Diagnostic Interview CIDI) criteria of a Major Depressive Episode—in the context of a unipolar major depressive disorder or bipolar disorder- and referred for ECT-treatment, are asked to participate in the cohort and, in case of occurrence of inter-ictal delirium, in the trial (22–24). Before inclusion, they provide a written informed consent form. The presence of an inter-ictal delirium is examined with the Confusion Assessment Method (25) (CAM) and/or Mini Mental State Examination (26) (MMSE), administered by a trained research assistant. For a diagnosis of inter-ictal delirium, patients need to have a CAM score indicating delirium or a drop in MMSE-score of at least 4 points as compared to baseline. The evaluation of inter-ictal delirium was done during regular screening once weekly, preferably in the middle of a week, 1 day (and maximum 2 days) after ECT. This timing was chosen to (1) select persistent cases of delirium, and hence not to include mild disorientation or transient delirious episodes immediately post-ECT; (2) to increase feasibility by avoiding cognitive testing during weekends and (3) to diminish the learning effect of repeated cognitive testing. Once delirium was diagnosed, an evaluation was done by the clinician to indicate whether it was needed to further exclude possible causes of delirium.

### Exclusion criteria

Exclusion criteria for participation in the cohort study are comorbid somatic conditions that are a relative contraindication for ECT, according to the prevailing Dutch ECT-guidelines, e.g., recent (within 3 months) myocardial infarction or cerebrovascular accident, a diagnosis of neurocognitive disorder, insufficient command of the Dutch language and prior participation in the cohort study (27). Additional exclusion criteria for participation in the trial are: diagnosed (treatable) somatic causes of delirium other than ECT (e.g., urinary tract infection) and contra-indications for rivastigmine use, such as bradycardia or atrio-ventricular (AV) conduction disorder (first degree AV-block excluded), current use of a AChI or a known allergic or adverse reaction to rivastigmine. As it is a cross-over trial, each cross-over patient serves as his or her own control. Since large, intercurrent changes in individual patients' situation hamper a cross-over comparison, patients who switch from (right) unilateral electrode placement (RUL) to bilateral electrode placement (BL), or individuals who receive dose adjustments of antipsychotics during the trial will be marked as drop-outs. Notably, participation in the cohort can be continued when patients drop out due to trial specific exclusion criteria.

### Recruitment

When ECT is indicated (for depression) and the patient is referred by the treating psychiatrist, the patient is subsequently informed on the study and protocol. This information is provided by the treating physician or a trained research assistant, both orally and with a patient information letter. Next, the patient is given 24 h to consider participation in the study, before written informed consent (for the cohort and if eligible for the trial) is obtained from each patient or legal representative (available upon request, notably the informed consent form is written in the Dutch language). The consent can be withdrawn at any time without citing reasons and without any consequences for the treatment. There is no financial incentive for the patient. The duration of inclusion is estimated at ~4 years. Promotional materials, including a folder and posters, have been developed and are available upon request.

### ECT procedure

ECT is given twice weekly. Electrode placement is decided by the ECT team, in accordance to Dutch ECT-guidelines (27). A course starts preferably with right unilateral stimulation (RUL), but bilateral stimulation (BL) is also allowed. Seizures are induced using the Thymatron system IV, Somatics, Lake Bluff, Illinois, USA, (maximum energy 200%, 1,008 millicoulombs) or the SpeECTrum 5000 Q, MECTA corp., Tualatin, Oregon, USA (max energy 200 J, 1,152 millicoulombs). Considering the interaction with seizure quality, we aim to discontinue lithium and other mood stabilizers at least 3 days before ECT starts. For the same reason, we aim to taper down benzodiazepines to a maximum of 30 milligrams oxazepam or 10 milligrams diazepam 3 days prior to the start of ECT. Antipsychotics and antidepressants may be continued and their use is recorded at each ECT-session. Anesthesia is induced with intravenous etomidate (±0.25 mg/kg) or propofol (±1 mg/kg) and muscle relaxation with succinylcholine (1–2 mg/kg). Patients are ventilated with 100% oxygen during the procedure in order to obtain hyperoxygenation status. The oxygen saturation (SpO_2_) level is kept above 95% in the pre-treatment phase. The ECT team performs a stimulus-titration procedure in the first session to determine the seizure threshold. The electroconvulsive treatment goal is set at six times the seizure threshold, brief pulse (0.5–1.0 ms), for RUL ECT, and 1.5–2.5 times seizure threshold for BL ECT. The ECT course is terminated when the patient reaches remission, defined by a Montgomery Åsberg Depression Scale score (28) (MADRS) of ≤ 10/60 during two consecutive weeks, when intolerable side-effects occur, or when no improvement occurs for 2 weeks after a full course of ECT is provided [maximum of six sessions of RUL ECT and six sessions of BL ECT (in total 12 sessions)].

### RECALL trial: Treatment allocation, intervention, and blinding

After inclusion in the trial, randomization is organized with a two by two Williams design (29). Block randomization is performed by the pharmacy department of the Amsterdam University Medical Center—location VUmc, with stratification for each site. Patients are randomly assigned to either the rivastigmine-followed-by-placebo group, or the placebo-followed-by-rivastigmine group. Each treatment arm lasts for four consecutive ECT sessions (Figure 1). Except for the unblinded nurse or research assistant (i.e., nurse or research assistant who is not involved in other parts of the study, nor in clinical practice, but only applies the patches), all other study members are blinded. “Trial treatment randomization lists” with codes are provided by an independent pharmacist and will only be available for the unblinded nurse or research assistant who applies the patches. Rivastigmine patches delivering 4.6 mg/24 h (Hexal AG, Holzkirchen, Germany) and Hansaplast® placebo patches (of similar size) are labeled in accordance with EU GMP annex 13 regulations and supplied to the sites. To improve and monitor adherence to the trial protocol, drug accountability lists are filled in. The Tmax of rivastigmine patches is 10–16 h, therefore the application of the rivastigmine (and placebo) patches is done the evening prior to ECT. The patch is removed after 24 h. As previously mentioned, during the trial, co-medication is allowed and there are no restrictions to clinical care as usual. As specified in the exclusion criteria however, patients with adjustments in antipsychotic medication or in electrode placement, are excluded from the trial. Benzodiazepines are allowed (e.g., in cases of profound agitation or insomnia). All changes in medications during the trial are registered after every ECT-session. Prior to each ECT-session, side-effects of rivastigmine are assessed, and in case of serious side-effects (e.g., gastrointestinal adverse reactions such as severe nausea), the participation in the trial will be stopped and rivastigmine discontinued. In case of persistent side-effect after discontinuation, the clinician will treat the patient according to the clinical picture (e.g., anti-emetics in case of nausea).

### RECALL trial: (Emergency) unblinding procedure

Unblinding will take place after ending the treatment period by the research nurse. Emergency unblinding may occur on an individual basis for safety reasons—such as the occurrence of a (Serious) Adverse Event [(S)AE] or Suspected Unexpected Serious Adverse Reaction (SUSAR). The medical team will contact the local investigator, through which the decision to unblind will be made. For emergency unblinding, envelopes are available at the medication storage room at the in-patients' ward, kept separately from the patches. The sealed envelope can be opened by anyone on the clinical team. The reason and time of unblinding will be documented in the study files.

### Outcome measures

Table 1 shows a full summary of patient characteristics and outcome measures, including the time of collection.

**Table 1:** Collection of patient characteristics and outcome measures in the RECALL trial and cohort.

	**Baseline**	**Regular care**		**Trial**							**Regular care in cohort**		
		**in cohort**									**after finishing the trial**		
	**Baseline**	**Max 90 minutes after ECT**	**Once a week during ECT** ^∧^	**Day before first ECT**	**Max 90 minutes after first ECT**	**Max 90 minutes after 2**^nd^ **ECT**	**Day after 2**^nd^ **ECT**^∧^	**Max 90 minutes after 3**^rd^ **ECT**	**Max 90 minutes after 4**^th^ **ECT**	**Day after 4**^th^ **ECT**^∧^	**Max 90 minutes after ECT**	**Once a week during ECT** ^∧^	**1 week after stop ECT**
Time	Ta	Tb		T0	T1	T2	T3	T4	T5	T6	Tb		
Socio-demographics	X												
*General clinical-demographics*													
Medical history	X												
Substance use	X												
Medication use	X	X		X	X	X		X	X	X	X		X
ATHF	X												
BMI	X												
Waist circumference	X												
Blood pressure	X												
*Physical activity and frailty*													
Frailty index	X												X
SPPB	X												X
Physical activity*	X												
Vision and hearing ability	X												
*Depression related clinical characteristics*													
BAI^a^	X												
GDS^a^	X												
AES^a^	X			X			X			X			X
MADRS^a^	X		X				X			X		X	X
CORE	X		X				X			X		X	X
*Neurocognitive characteristics*													
Battery of neuropsychological tests**	X												X
Self reported cognitive decline	X												X
IQCODE	X												X
(f)MRI	X												
EEG	X												
*Laboratory characteristics*													
Blood / urine sample	X			X									X
*Primary outcome measure*													
DRS-R-98				X	X	X	X	X	X	X			
*Secondary outcome measures*													
MMSE	X		X				X			X		X	X
Letter fluency	X			X			X			X			X
Clock Drawing Test	X			X			X			X			X
Reorientation time		X			X	X		X	X		X		
RASS		X			X	X		X	X		X		
CAM		X	X										
ECT characteristics		X			X	X		X	X		X		
Rivastigmine side-effects				X	X	X		X	X	X			

*ATHF, Antidepressant Treatment History Form; BMI, Body Mass Index; SPPB, Short Physical Performance Battery; BAI, Beck Anxiety Inventory; GDS, Geriatric Depression Scale; AES, Apathy Evaluation Scale; MADRS, Montgomery Åsberg Depression Scale; IQCODE, Informant Questionnaire on Cognitive Decline in the Elderly; DRS-R-98, Delirium Rating Scale-Revised-98; MMSE, Mini-Mental State Examination; RASS, Richmond Agitation Sedation Scale; CAM, Confusion Assessment Method*.

∧ *The day of measurement is Wednesday, as ECT is done twice weekly*.

* *Physical activity includes assessment of mobility, International Physical Activity Questionnaire (IPAQ), and self-reported activities of daily living (ADL)*.

** *Stroop Color Word Test, 15-Words Test or Auditory Verbal Learning Test, Trail making Test, Kopelman Autobiographic Memory Interview, and Visual Association Test*.

#### Primary outcome measure

During the trial, the effect of rivastigmine on inter-ictal delirium is examined through the Delirium Rating Scale-Revised-98 (DRS-R-98) (30). It was demonstrated that the DRS-R-98 is sensitive, specific, reliable, and easy to use for the assessment of severity of a delirium (30). We will compare delta-scores of the DRS-R-98 computed over the period of rivastigmine-use and placebo (T3-T0 and T6-T3, depending on whether the patients start with rivastigmine or placebo). This is assessed by a trained, blinded research nurse once weekly.

### Secondary outcome measures

Additional cognitive function tests are used to further specify the cognitive deficits, namely the Mini Mental State Examination (26) (MMSE), letter fluency or Controlled Oral Word Association Test-FAS (31), the Clock Drawing Test (32), reorientation time (33, 34), the Richmond Agitation Sedation Scale (35) (RASS) and the Confusion Assessment method (25) (CAM). The MMSE is a screening instrument for measuring the severity of cognitive impairment with good interrater and test-retest reliability (25). The Controlled Oral Word Association Test (COWAT) is widely used in clinical neuropsychology and is a measure of language and executive function. The patient is asked to name as many words as possible with the assigned letter. Patients cannot use names or numbers and cannot use words with different tenses or endings once the root word has been given. Psychometric properties were similar in the Dutch-version as compared to the original version, with reliability-scores of 0.8 (31). The COWAT is specifically chosen as an addition to the MMSE to test executive functioning. The Clock-Drawing Test (CDT) is another widely used cognitive screening tool of executive function, visuospatial abilities, motor execution, attention, language comprehension, and numerical knowledge, with good reliability and validity in patients with cognitive impairment (36). Patients are verbally asked to draw an analog clock, including all of the numbers, and set to a specified time (e.g., 10 min past 11 am). In order to diminish a learning effect of repetitive cognitive testing, the three-word recall test (included in the MMSE) and letter fluency differs in consecutive sessions. The early reorientation time—or postictal orientation recovery time—is the time until reorientation directly post-ECT, until a maximum of 90 min. It is a continuous assessment by the research nurse, asking the patient to state his / her name, location, age, date of birth and the day of the week. Four correct answers out of five questions is considered as adequate orientation (34). When unable to answer correctly within 90 min, a score of 100 min will be noted. The reorientation time is an easy test to administer. Duration of postictal reorientation has been associated with the extent of retrograde amnesia in the past (34). Sedation or agitation status is assessed by the Richmond Agitation Sedation Scale (RASS) (35). The RASS has excellent interrater reliability and criterion-, construct-, and face validity (37). It takes around 30 s to administer. The Confusion Assessment Method is used as an outcome measure in the cohort to define the amount of patients with a probable delirium in the cohort. It takes ~2 min to administer. For a diagnosis of delirium by CAM, the patient must display: (1) presence of acute onset and fluctuating discourse, and (2) inattention, and either (3) disorganized thinking or (4) altered level of consciousness. The CAM has good sensitivity (94–100%) and specificity (90–95%) and the interobserver reliability rates are high (0.81–1.00) (25).

Furthermore, in order to investigate the safety and effect of rivastigmine on ECT-parameters and anesthesia, we measure blood pressure, peak heart rate, seizure length (EEG and motor duration in seconds), postictal suppression index (in %) and seizure threshold. Apart from the postictal suppression index and seizure length measured by the Thymatron IV (unavailable at sites with the spECTrum), all variables are recorded by a blinded anesthesiologist. Next, side-effects of rivastigmine are evaluated twice weekly (by a research assistant) using a four-point side-effects scale with the five most common side-effects of rivastigmine (nausea, vomiting, diarrhea, abdominal pain, allergic skin reaction), and an open question for additional side-effects.

#### Patient characteristics

In addition to the outcome measures, various patient characteristics are assessed, including socio-demographics, physical-, clinical-, and neurocognitive characteristics. All variables are collected by a trained research nurse or assistant, unless otherwise specified.

Socio-demographics include age, gender, partner status, current living situation, level of education (total years of education), all self-reported.

Physical characteristics include Body Mass Index (BMI), waist circumference (centimeters), blood pressure (mmHg), vision- and hearing, dental condition, smoking status (current, former, never), alcohol use (number of drinks per week), activities of daily living, physical activity, International Physical Activity Questionnaire (38), Short Physical Performance Battery (39) and frailty status (40). Vision and hearing ability is measured with self-reported questions on the quality of vision and hearing and questions on the presence of glasses or hearing devices. Dental condition is measured with a question on the presence of dentures. Level of activities of daily living (ADL) is measured through 6 questions on the ability of the patient to (1) shower, (2) dress up, (3) go to the bathroom, (4) transfer from bed to chair, (5) eat, and whether the patient (6) uses incontinence material. Physical activity is measured by a 16-item questionnaire on self-reported mobility (i.e., questions on the risk of falls, need of walking aids, maximum walking distance, ability to climb stairs, cycling) (available upon request). The International Physical Activity Questionnaire (IPAQ) is a validated questionnaire in older adults for assessing physical activity, measured in metabolic equivalent minutes (MET-minutes). The MET-minutes are calculated through assigning a ratio of energy expenditure during an activity (as compared to energy expenditure in resting state), multiplied by the amount of minutes these activities are performed per week (38). The Short Physical Performance Battery (SPPB) is a reliable and valid score to assess lower extremity function. The SPPB sums up the scores of three timed performance tests: the tandem balance test; the walking speed; and the repeated rising up from the chair-test. It gives important information on actual lower extremity function and is a predictor and outcome which can be used for physical decline (41). The prevalence of frailty using Fried's phenotype will be assessed (40). This phenotype consists of 5 variables: unintentional weight loss, muscle weakness, self-reported exhaustion, poor endurance, and low activity level. Patients will be classified as non-frail (0 variables present), pre-frail (1-2 variables present) or frail (3 or more variables present). Unintentional weight loss is measured with a positive response to the CIDI question about unwanted weight loss (with a minimum of 1 kg/week or a BMI ≤ 18.5 kg/m^2^). Muscle weakness is measured through hand grip strength [for a detailed description see Penninx et al. (42)]. Self-reported exhaustion is measured according to two statements of the Center for Epidemiologic Studies-Depression scale (43) (CES-D), namely (a) I felt that everything I did was an effort, and (b) I could not get going. If at least one condition is present for 3 days or more during the last week, the criterion is positive. Poor endurance will be defined with the walking speed test from the SPPB. Low physical activity level will be defined with the IPAQ.

Clinical characteristics include somatic and psychiatric (family) history, presence of chronic diseases, medication use, treatment resistance, Geriatric Depression Scale (44), Montgomery Åsberg Depression Rating Scale (28), Beck Anxiety Index (45), Apathy Evaluation Scale (46), and the CORE (47). Somatic and psychiatric (family) history is self-reported and checked by examining clinical records. Standardized questions for chronic diseases are used, which include chronic pulmonary disease, cardiovascular disease, diabetes, cerebrovascular disease, osteoarthritis, rheumatoid arthritis and cancer. These categories were chosen based on prevalence rates (48). In addition, patients are asked whether they have any other chronic diseases (defined as a disease of which symptoms and/or treatment had been present for at least 3 months). Current medication use, medication changes during ECT course and treatment resistance through the Antidepressant Treatment History Form: Short Form (49) (ATHF-SF) are noted by the treating physician. The 15-item self-reported Geriatric Depression Scale (GDS) is a valid and reliable scale for differentiating depressed from non-depressed patients (44). The 10-item Montgomery Åsberg Depression Rating Scale (MADRS) is a widely used scale to measure the severity of depressive symptoms with high interrater reliability and good validity (50). The 21-item Beck Anxiety Index (BAI) helps to discriminate between anxiety and depression and measures the severity of anxiety with high internal consistency and test-retest reliability (45). Apathy is measured with the Apathy Evaluation Scale—clinician version (46) (AES-C). The AES-C is a well-known scale with good reliability and validity (46). Severity of psychomotor symptoms and the probability of melancholia is measured by the CORE, an 18-item observation scale subdivided into three subscales (retardation, agitation and non-interaction) (47). It has high inter-rater reliability and good validity for psychomotor disturbance in depressed persons (51).

Neurocognitive characteristics include the Stroop Color and Word Test (52), the 15-words Test (53), the Trail Making Test (54), Kopelman Autobiographical Memory Interview (55), the Visual Association Test (56), self-reported cognitive decline and the Informant Questionnaire on Cognitive Decline in the Elderly- Short Form (57) (IQCODE-SF). The Stroop Color and Word Test is a neuropsychological test extensively used to assess the ability to inhibit cognitive interference, working memory and speed of visual search (58). The 15-words test, or Auditory Verbal Learning Test, is a test used to assess attention, memory, and learning ability. The Trail Making Test A and B is used to assess executive functioning, visual search and scanning and speed of processing. Kopelman Autobiographical Memory Interview is used to measure autobiographical and semantic memory. The Visual Association Test is used to measure signs of anterograde amnesia through visual associated learning. Self-reported decline is measured with the CIDI questionnaire, section E (22). The IQCODE-SF is a 16-item questionnaire for caregivers that is used for screening and evaluation of cognitive decline and dementia in patients.

In a subset of patients—those derived from GGZ inGeest and OLVG hospital and whom provided informed consent– we conduct an electroencephalogram (EEG) and (Functional) Magnetic Resonance Imaging [(f)MRI] at baseline. EEG assessment is a non-invasive, widely available, cheap and objective measure for brain functioning, capable of detecting signs of delirium or vulnerability of the brain associated with neurocognitive disorder (59, 60). On MRI, the presence of white matter hyperintensities [Fazekas score (61)], cortical atrophy [Pasquier or GCA score (62)] and medial temporal lobe atrophy [Scheltens or MTA score (63)] atrophy are rated. In order to obtain insight in brain activity as well, MRI scans will be extended by 15–20 min to derive functional imaging (fMRI).

Laboratory characteristics include inflammation markers (e.g., high sensitive CRP, interleukin-6), s100-beta, the regular laboratory tests done in a comprehensive geriatric assessment (such as complete blood count, basic electrolyte and metabolic panel, kidney and thyroid functioning, vitamin and lipid status) and urine samples (64, 65). S100-beta is a calcium-binding protein expressed by astrocytes, associated with delirious states and cognitive decline during ECT (66, 67). Urine morning samples are collected for the assessment of several biomarkers which have shown to be involved in the prediction, diagnosis and evaluation of antidepressant treatment in recent studies (68, 69). The final selection of biomarkers of interest will be done at time of laboratory processing, depending on state-of-the art laboratory techniques, possibly including additional inflammation markers and/or neurotransmitters.

### Sample size calculation

We estimated that in our vulnerable study population, prevalence rates of delirium and profound cognitive decline are ~20%, based on a previous study (11). In order to detect a reduction on the DRS-R-98 of 4 points (SD ± 7), at 80% power with α = 0.05, 120 delirious patients should be included in a non-cross-over trial. These patients (*n* = 120) should be identified during regular ECT (cohort study). Since the estimated prevalence of inter-ictal delirium is ~20%, the cohort should include in total at least 600–1,000 persons in order to select 120 patients. This is not possible, due to limited availability of resources. Therefore, a cross-over design will be used. A cross-over design requires fewer patients than a non-cross-over trial to achieve similar power. With an estimated correlation between intra-individual measurements of *r* = 0.50, a cross-over trial requires only 25% of the number of patients (70). Hence, a total of 30 patients provide sufficient power for the trial. With an estimated dropout of 20%, 38 patients need to be included to obtain sufficient power for the trial. We aimed at a probability of 75% to include 38 delirious patients in the trial, which would amount to 210 patients in the cohort. Eventually, with an additional 20% margin of safety due to estimated prevalence, we decided to set the inclusion goal at 250 patients for the cohort, to achieve a sample of 30 delirious patients completing the trial. However, during the first months of data-collection, the prevalence of inter-ictal delirium was noticeably higher, namely ~25%. Hence, in an amendment, the goal of inclusion in the cohort was set back to 150 patients.

### Data collection, management, and analysis

#### Data collection and retention

All assessors are trained in the questionnaires or instruments they assess. In addition, they are trained to motivate patients to complete follow-up. All training sessions for the Recall study are recorded in a training log.

#### Data management

The acquired data and examination results will be entered into an electronic case record form (eCRF), stored on a laptop, only accessible by members of the research team. Where it is necessary to be able to trace data to an individual subject, a subject identification code list will be used to link the data to the subject. This code list will be stored at the department of psychiatry Amsterdam University Medical Center—location VUmc. The key to the code will be safeguarded by the data management in case the data or the blood samples are kept for a longer period of time. The handling of personal data is in accordance with the Dutch Personal Data Protection Act (in Dutch: De Wet Bescherming Persoonsgegevens).

#### Data analysis

Significance will be set at *p* < 0.05 for all statistics and 95% confidence intervals will be presented. Intention to treat analyses will be conducted. Descriptive baseline statistics will be generated for the patients included in the trial. As the trial is a patient cross-over design, no comparison or correction for baseline variables will be done.

To estimate the efficacy of rivastigmine on inter-ictal delirium in the RECALL trial, delta scores of the DRS-R-98 will be used. These delta scores will be computed over the period of rivastigmine (intervention, 2 ECT sessions, 7 days) and placebo (comparison, 2 ECT sessions, 7 days) separately. For further details, see section Primary outcome measure. Analysis of Variance (ANOVA) by a mixed model for repeated measures, with treatment (rivastigmine vs. placebo) as a within effect and sequence of treatment (arm A vs. arm B) as a between factor, will be performed. Additionally, the association between rivastigmine and (delta-scores of the) DRS-R-98 will be examined in a traditional regression model, with putative effect modifiers, confounders and determinants.

Cox's regression analyses will be conducted in the RECALL cohort, with outcome defined as the (time to) occurrence of inter-ictal delirium measured by a positive CAM and/or a decline of MMSE of 4 points, adjusted for putative effect modifiers, confounders and determinants. Additionally, putative patterns of cognitive decline will be identified by data-driven methods (e.g., latent-class analysis), based on cognitive outcome measures. Future analyses in the data will depend on appropriate research questions.

### Monitoring, auditing, and harms

#### Monitoring and auditing

A data monitoring committee has been installed, consisting of the Clinical Research Bureau (CRB) of VUmc, Amsterdam (currently Amsterdam University Medical Center). This CRB is independent from the sponsor and has no competing interests. In summary, the CRB monitors whether the rights and well-being of the respondents are protected; whether data from the research is reported correctly and fully verifiable in source documents; and whether the execution of the study is in accordance with the protocol or amendment(s) approved at that time, with Good Clinical Practice (GCP) and with the relevant legal requirements. In addition, monitoring visits at all Dutch sites are conducted shortly after the start of the study, and once yearly during the study. Audits on trial conduct are performed at random by the Amsterdam Public Health Research Institute. Considering the low risk of rivastigmine, as a long-term registered drug, an additional Data and Safety Monitoring Board was not installed. Likewise, interim analyses will not be conducted. Notably, all documents, including Patients' Information Folder (PIF) and Patients' Informed Consent forms (IC), and protocols for MRI and EEG measurements, blood and urine collection, laboratory evaluation and storage and data management plan (including information on data entry, coding, security and storage) are available upon request.

#### Harms

Adverse events are defined as 'any undesirable experience occurring to a subject during a clinical trial, whether or not considered related to the intervention'. Therefore, we decided to note only adverse events in the trial, and not during regular ECT-treatment (cohort-study). All adverse events reported spontaneously by the subject or observed by the investigator or his staff are recorded. All serious adverse events, defined by the Central Committee on Research involving Human Subjects (CCMO), that occur in any of the participating centers are reported immediately to the principal investigator, who will report to the medical ethical board of the VUmc. The local investigators at each collaborating center are responsible for immediate reporting to the principal investigator.

## Discussion

The RECALL study aims to provide insight into the effects of rivastigmine on inter-ictal delirium, and to study the determinants and subtypes of cognitive side-effects of ECT in older persons. For this purpose, the design consists of a prospective cohort study in older patients treated with ECT for depression, primarily aiming to identify patients eligible for the cross-over trial to examine rivastigmine as a treatment for inter-ictal delirium. The prospective cohort is efficiently used to provide insight into cognitive side-effects and their determinants. We hypothesize that rivastigmine can prevent the excessive post-ECT drop in acetylcholine, and through this mechanism, reduce inter-ictal delirium severity. To our knowledge, the RECALL study will be the first clinical trial in patients with inter-ictal delirium during ECT.

The RECALL study will provide us with a large selection of putative determinants and outcome measures of ECT-related cognitive side-effects. Since these determinants are measured at baseline, their association with the onset of inter-ictal delirium can be assessed. Furthermore, the wide array of cognitive measures could enable detection of ECT-related cognitive side-effects. To date, studies on cognitive side-effects during ECT are often hampered by limited assessment of (putative predictors of) ECT-related cognitive side-effects. In addition, it enables detailed description of the study sample, facilitating generalizability of findings.

The cross-over design is chosen for three specific reasons; (1) to reduce the influence of confounding covariates, as each crossover patient serves as his or her own control; (2) to enable all trial patients to benefit from the study medication; and (3) most important, feasibility, as cross-over designs require fewer patients than non-cross-over designs to achieve sufficient power (71). The feasibility was the major argument for the design team as this is a population that is particularly hard to study. It is difficult to include patients due to less motivation which is a symptom of their disease, and—in case of legal incapability- doubts from the legal representative (e.g., family) to agree to an additional extensive battery of tests during the treatment. In addition, as in all studies on depression, we predict that the anhedonia and indecisiveness, core symptoms of depression, cause difficulties for patients to stay motivated during the entire study, resulting in missing data. The cross-over design allows this study to achieve sufficient power with 30 delirious patients completing the trial. A potential bias in cross-over designs may be “carry-over” effects. However, rivastigmine has a short elimination time [T1/2 patch = 3.4 h (72)], therefore a minimal “wash-out” period of 1 day is deemed sufficient. After removal of the patch, rivastigmine is eliminated for more than 99% within 24 h—or before the next ECT session is done. Furthermore, transdermal rivastigmine was chosen over other AChI due to reported superiority on gastro-intestinal side-effects and similar expected efficacy (73–75).

A limitation of our study is the possibly invalid use of the MMSE as an inclusion measure for patients with an inter-ictal delirium. It might be improper, as it is previously not validated as a tool for the diagnosis of inter-ictal delirium, or any other delirium (76). The same goes for the MMSE as a measure of delirium severity. However, we claim, based on our clinical experience, that inter-ictal delirium does not always behave as a “regular” delirium and therefore does not always meet the criteria for delirium as measured with the CAM. For example, hallucinations are often lacking, and the patients usually suffer from clouding of consciousness, persistent disorientation or reduced speed of processing. Hence, by screening for inter-ictal delirium using the CAM only, eligible patients for the trial might be missed.

In short, this paper provides the study protocol of the first clinical trial on interictal delirium, embedded in a cohort study that extensively measures ECT-related cognitive side-effects and their putative determinants. These extensive (outcome) measures facilitate future research, and may contribute to a future consensus on cognitive testing during ECT. Most importantly, this study may provide evidence for treatment of inter-ictal delirium with rivastigmine.

## Ethics Statement

The studies involving human participants were reviewed and approved by Medical-Ethical Board of VU University Medical Center and the Central Committee on Research involving Human Subjects (CCMO). The patients/participants provided their written informed consent to participate in this study.

## Author Contributions

DR and MH initiated the study and are grant holder. TF, MH, and DR drafted the manuscript. EvE, JB, AV, AvdL, NvdV, AD, ME, PS, FB, HS, RK, SL, AB, and MS helped with the design and implementation of study. PB provided expertise in clinical trial design (handling medication and randomization format). EvE, AV, AvdL, PS, FB, HS, and RK were assigned as local lead investigator and were responsible for recruitment and data collection, and organization of the study in participating centers. All authors contributed to reviewing the manuscript, and approved the final manuscript.

## Funding

ZonMW goed gebruik geneesmiddelen (https://www.zonmw.nl/nl/); project number 848015012: no influence on any aspect of the study; only monitoring progress. Amsterdam University Medical Center: delegation of all study responsibilities to principal investigator, including study design, data collection, management, analysis, and interpretation of data; writing of the report; and the decision to submit the report for publication.

## Conflict of interest

The authors declare that the research was conducted in the absence of any commercial or financial relationships that could be construed as a potential conflict of interest.

## Publisher's Note

All claims expressed in this article are solely those of the authors and do not necessarily represent those of their affiliated organizations, or those of the publisher, the editors and the reviewers. Any product that may be evaluated in this article, or claim that may be made by its manufacturer, is not guaranteed or endorsed by the publisher.
